# Population genomics of the grapevine pathogen *Eutypa lata* reveals evidence for population expansion and intraspecific differences in secondary metabolite gene clusters

**DOI:** 10.1371/journal.pgen.1010153

**Published:** 2022-04-01

**Authors:** Cristobal A. Onetto, Mark R. Sosnowski, Steven Van Den Heuvel, Anthony R. Borneman

**Affiliations:** 1 The Australian Wine Research Institute, Adelaide, Australia; 2 South Australian Research and Development Institute, Adelaide, Australia; 3 School of Wine, Food and Agriculture, The University of Adelaide, Adelaide, Australia; University College Dublin, IRELAND

## Abstract

Eutypa dieback of grapevine is an important disease caused by the generalist Ascomycete fungus *Eutypa lata*. Despite the relevance of this species to the global wine industry, its genomic diversity remains unknown, with only a single publicly available genome assembly. Whole-genome sequencing and comparative genomics was performed on forty Australian *E*. *lata* isolates to understand the genome evolution, adaptation, population size and structure of these isolates. Phylogenetic and linkage disequilibrium decay analyses provided evidence of extensive gene flow through sexual recombination between isolates obtained from different geographic locations and hosts. Investigation of the genetic diversity of these isolates suggested rapid population expansion, likely as a consequence of the recent growth of the Australian wine industry. Genomic regions affected by selective sweeps were shown to be enriched for genes associated with secondary metabolite clusters and included genes encoding proteins with a role in nutrient acquisition, degradation of host cell wall and metal and drug resistance, suggesting recent adaptation to both abiotic factors and potentially host genotypes. Genome synteny analysis using long-read genome assemblies showed significant intraspecific genomic plasticity with extensive chromosomal rearrangements impacting the secondary metabolite production potential of this species. Finally, k-mer based GWAS analysis identified a potential locus associated with mycelia recovery in canes of *Vitis vinifera* that will require further investigations.

## Author summary

Eutypa dieback of grapevine, caused by the Ascomycete fungus *Eutypa lata*, is responsible for significant economic losses to the wine industry. Despite the worldwide prevalence of this pathogen, its genomic diversity remains unknown, with only a single publicly available genome assembly. This knowledge gap was addressed by performing whole-genome sequencing of 40 *E*. *lata* isolates sourced from different hosts and geographical locations around Australia. Investigation of the genetic diversity of this population showed a high degree of gene-flow and sexual recombination as well as demographic expansion. Through the inspection of signatures of selective sweeps, repeat-mediated chromosomal rearrangements, and pan-genomic elements, it was shown that this species has a highly dynamic secondary metabolite production potential that could have important implications for its pathogenicity and lifestyle. In addition, application of a k-mer based GWAS methodology, identified a potential locus associated with the growth of this species within canes of *Vitis vinifera*.

## 1. Introduction

Eutypa dieback of grapevines is responsible for significant economic losses to the wine industry worldwide [1–3]. The fungal disease is caused by the Ascomycete *Eutypa lata* [1], which can affect a wide variety of woody plant species including grapevine, apricot, cherry, olive, peach and walnut [1–4]. Disease is generally spread by wind-dispersed ascospores infecting fresh pruning wounds. After landing on a suitable wound, *E*. *lata* ascospores rapidly germinate and begin colonizing the xylem vessels of the host [1]. The mycelium slowly spreads through the wood tissue, colonizing spurs, cordon and trunk, and eventually causing death of the grapevine [5].

Common symptoms of infection include wood necrosis observed as wedge-shaped cankers, leaf chlorosis and tattering of leaf margins, and stunting of shoots [1,6,7]. Foliar symptoms are thought to be caused by acetylenic phenol and chromene metabolites, which are produced by *E*. *lata* in the infected wood and then translocated to the foliage via the plant vascular system [8–10].

Variability in pathogenesis and disease susceptibility has been recorded between both strains of *E*. *lata* [6] and cultivars of *V*. *vinifera* [11], which are attributed to differences in the production of secondary metabolites [8] and xylem morphology and lignin composition of the wood, respectively [12,13]. Furthermore, analysis of the single draft genome available for this species revealed a large diversity of plant cell wall degrading enzymes and secondary metabolite clusters [14,15] that may explain the diversity of hosts infected by *E*. *lata*.

*E*. *lata* is considered a generalist fungus, regularly reported in continents where grapevines and specific *Prunus* species are cultivated, including Europe, North America, Australia, and South Africa [16]. Microsatellite based investigations of the genetic diversity of the *E*. *lata* population demonstrated high levels of gene flow between isolates and a lack of association between specific genotypic groups and either host or geographic location [17–19]. This lack of population structure has been hypothesised to arise from regular genetic reshuffling through sexual recombination and frequent immigration among hosts and geographic locations by spore dispersal and human-mediated transport of infected material [17,18].

In Australia, *E*. *lata* has been recognised as a major agricultural pathogen for over 60 years [20] and is suggested to have been introduced into the country from Europe through the transport of infected plant material [18]. Despite the relevance of this species to the Australian wine industry, the genomic diversity of the Australian *E*. *lata* population remains unknown. While microsatellite-based investigations have shed light on the global genetic diversity of this species [17,18], this technique has well documented limitations for the study of population genetics [21]. Additional genome sequencing efforts are necessary to understand the genome evolution of this species, including adaptation, population size and structure as well as the genetic determinants of pathogenicity. In this study we address this knowledge gap by performing whole-genome sequencing and population genomic analyses of 40 *E*. *lata* isolates obtained from different hosts and geographical locations around Australia, including four isolates sequenced using long-read nanopore technology.

## 2. Material and methods

### 2.1. DNA extraction and genome sequencing of *E*. *lata* isolates

Forty *E*. *lata* isolates were obtained from the South Australian Research and Development Institute (SARDI) collection (S1 Table) from which 35 have been previously published and phenotyped [6,7,22,23]. Details on isolation source are available in S1 Table. For short-read shotgun DNA sequencing, isolates were grown on Potato Dextrose (PD) broth (Sigma, Australia) for 10 days at 22°C after which samples were pelleted by centrifugation. DNA extraction from pellets was performed using a MagAttract Microbial DNA Kit (Qiagen, Australia) and a Precellys Evolution Homogenizer (Bertin, France) (30 s, 4500 rpm). Library preparation and sequencing was performed in the Ramaciotti Centre for Genomics (University of New South Wales, Sydney, Australia). Sequencing libraries were prepared using the Illumina DNA library kit and sequenced with an Illumina NovaSeq 6000 using 2 x 150 bp chemistry on a SP flowcell.

For long-read sequencing using nanopore technology, mycelium pellets were thoroughly squeeze-dried using a paper mesh, transferred into a mortar, and then frozen by directly pouring liquid nitrogen on top of the samples. Samples were then ground into a fine powder and transferred into 1.5 mL tubes already placed in dry ice. High molecular weight DNA was extracted directly from these tubes using a Gentra Puregene Yeast/Bact DNA extraction kit (Qiagen, Australia). Sequencing libraries were prepared using the SQK-LSK109 and EXP-NBD104 kits. Fast5 files were base called and demultiplexed using Guppy v. 4.5.3 (Oxford Nanopore Technologies, Oxford, UK) using the ‘hac’ model with a minimum quality score filtering of 7.

### 2.2. Genome assembly and annotation

Short-read assemblies of 36 *E*. *lata* isolates were performed with Spades v. 3.15.2 [24] and then filtered for contigs with a minimum size of 500 bp (S2 Table). Long-read assemblies of isolates TAS7, MA101, 511–17 and B003 (S2 Table) were assembled using Canu v. 2.1.1 [25] and Flye v. 2.8.3 [26]. Both assemblies were then combined using quickmerge v. 0.3 [27] to improve contiguity. Reads were then mapped back to assemblies and low coverage contigs were tagged and removed with Tapestry v. 1.0.0 [28]. Finally, assemblies were polished three times to correct for SNPs and indels using short-reads and Pilon v. 1.24 [29].

Gene prediction of genome assemblies was performed following the funannotate pipeline v. 1.8.7 [30], including Genemark-ES v. 4.68 [31], SNAP [32], Augustus v. 3.3.3 [33] and Glimmerhmm v. 3.0.4 [34] annotations trained using BUSCO v. 2 [35]. Repeats were identified using RepeatMasker v. 4.1.1 [36] and LTRharvest [37]. Functional annotations were performed using the UniProt database (2021_02), Interproscan 5 [38], Pfam v. 34.0 [39], antiSMASH v. 5.0 [40], SignalP v. 4.1 [41] and dbCAN v. 9.0 [42]. Assembly and annotation statistics for all isolates are available in S2 Table.

### 2.3. Genome synteny analysis

Prior to alignment, contigs from each assembly were reordered based on the reference assembly using Mauve v. 2.4.0 [43]. Alignments were performed between the long-read assemblies of isolates TAS7, MA101, 511–17 and B003 using Nucmer v. 3.1 [44] and coordinates obtained with the show-coords function. Synteny breakpoints associated with chromosomal rearrangements were queried against the predicted repeats using BEDTools v. 2.30.0 [45] and only repeats with a distance of < 2 kb from the synteny breakpoint were retained and manually inspected. Synteny breakpoints located within 50 kb of contig ends were masked to avoid highly repetitive telomeric repeat regions. For visualisation of the alignments, closely adjacent synteny blocks were smoothed and plotted using Circos v. 0.69 [46].

### 2.4. Phylogenetic and population genetics analyses

Illumina reads from 40 isolates (S1 Table) were mapped to the long-read genome assembly of *E*. *lata* strain TAS7 using Bowtie2 v. 2.3.4 [47] keeping only reads with a minimum mapping quality of 30. Duplicate reads were removed with Picard v. 2.18.4 [48]. Pileups were generated using SAMtools v. 1.8 [49] and variants were called using VarScan v. 2.3.9 [50] with a minimum variant allele frequency threshold of 0.4 and minimum supporting reads at position cut-off of 10. Consensus calls were obtained for all positions where SNPs were found in any of the samples and merged using BcfTools v. 1.8 [51], keeping only homozygous SNP calls.

A maximum-likelihood phylogenetic tree was constructed using IQ-TREE v. 2.1.2 [52] with the GTR+ASC model using the consensus SNP matrix filtered by calls present in all samples and with a minor allele frequency ratio (MAF) of 0.05. Linkage disequilibrium decay was estimated by calculating the correlation coefficient (*r*^2^) between pairs of loci present in the largest contig with a maximum distance of 100 kbp using vcftools v. 0.1.17 [53]. Values were averaged in 1 bp windows and plotted as a function of distance using R [54] and the ggplot2 package [55]. Tajima’s *D* was calculated in 100 kbp windows across all the genome using vcftools v. 0.1.17 [53] and the MAF unfiltered SNP matrix. Plotting of the MAF spectrum was performed in R [54] using the ggplot2 package [55] and the MAF unfiltered SNP matrix.

Pan-genome analysis was performed with predicted coding DNA sequences (CDS) of the short-read assemblies using the GET-HOMOLOGUES-EST pipeline [56] and OrthoMCL [57].

Screen for selective sweeps across the genome was performed using RAiSD v. 2.9 [58] and the *μ* statistics. The *μ* statistic relies on multiple signatures of a selective sweep via the enumeration of SNP vectors, including expected reduction of variation in the region of a sweep, shifts in site frequency spectrum (SFS) and emergence of localized LD patterns on each side of the beneficial mutation [58]. Prior to running the pipeline, missing regions in all samples as well as telomeric and centromeric repeats were masked to avoid inflated *μ* scores. The top 0.01% scored windows were then selected and genes withing these regions were extracted using BEDTools v. 2.30.0 [45].

### 2.5. Genome wide association analysis (GWAS)

GWAS analyses was performed using the kmerGWAS pipeline v. 0.2 [59]. The k-mer database was built with 2 x 150 bp Illumina reads using KMC v. 3 [60] with a k-mer size of 31 bp. The kinship matrix was calculated using EMMA [61] with a minor allele frequency of 0.02 and a minor allele count of 5. GWAS was performed using the previously published phenotype data of mycelium recovery for 25 *E*. *lata* isolates [6]. In Sosnowski, Lardner [6], this phenotype was determined by drilling *E*. *lata* containing agar plugs into the apex of rootlings of *V*. *vinifera*. Mycelial spread was assessed 24 months after inoculation by isolation at 5 to 10 mm intervals and confirmed using specific DNA markers. Long-nanopore reads from isolate B003 were queried for the presence of the top significantly associated k-mers and then mapped back to the long-read genome assembly.

### 2.6. Meta-transcriptomic analysis

Previously published RNA-seq data [62] of grapevines showing symptoms of Eutypa dieback (samples ED1-8) were combined and mapped using the splice-aware mapping software STAR v. 2.7.9a [63] to a multi-species closed reference genome described by Morales-Cruz, Allenbeck [62] with the replacement of *E*. *lata* isolate UCREL1 with the long-read assembly of *E*. *lata* isolate TAS7. High quality mapping reads to *E*. *lata* were retained and gene counts were performed using featureCounts v.2.0.0 [64]. Counts were normalised as transcripts per million (TPM) as previously described by Wagner, Kin [65].

## 3. Results and discussion

The genetic diversity of an Australian *E*. *lata* population was investigated through whole genome sequencing and SNP-based analyses. Forty isolates, sourced from different hosts and grape-growing geographical locations, were subjected to short-read sequencing (S1 Table). In addition to short-read sequencing, four of these isolates (S2 Table) were also sequenced using nanopore long-read technology to obtain contiguous *de novo* genome assemblies for SNP-based analyses and investigation of the presence of genomic architectural rearrangements.

**Fig 1:**
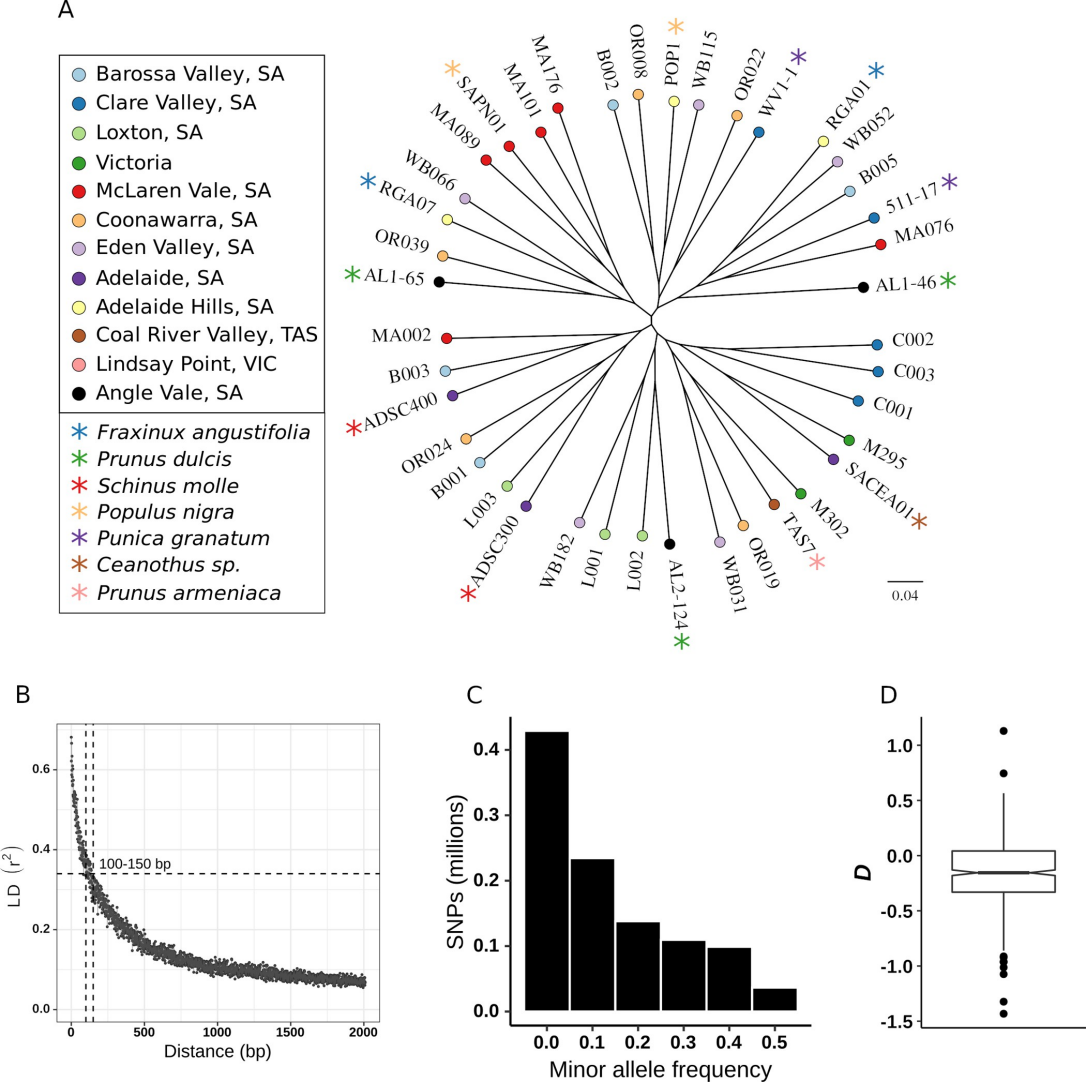
Genetic diversity within 40 Australian *Eutypa lata* isolates. A. Maximum-likelihood phylogenetic tree constructed from 740,941 single nucleotide polymorphisms (SNPs). Isolation location and host are indicated in the legend. Isolates without an asterisk (✱) were isolated from *Vitis vinifera*. B. Linkage disequilibrium (LD) decay on all bi-allelic SNPs present in the largest contig (6.7 Mbp). Data is presented as the squared correlation coefficient (r^2^) between pairs of SNPs averaged in 1 bp windows. Dotted lines indicate half of the maximum observed value and the corresponding physical distance. C. Minor allele frequency spectrum of SNPs present in all samples. D. Boxplot of Tajima’s *D* distribution calculated per 100 kbp windows.

To better understand the phylogeny of the Australian *E*. *lata* population, SNPs were identified between the forty isolates. After filtering, 740,941 SNPs were retained and used for phylogenetic reconstruction (Fig 1A). The absence of heterozygous SNPs confirmed all isolates were either haploids or homozygous diploids. Phylogenetic analysis showed no evidence of clonality (Fig 1A). Two sets of isolates sourced from the regions of McLaren Vale (isolates MA089, SAPN01, MA101, MA176) and Clare Valley (C001, C002 and C003) grouped into clades based on geographic location (Fig 1A). From these, isolates labelled MA were obtained from different grapevines within a single vineyard and isolates C from three grapevine varieties (Cabernet Sauvignon, Chardonnay and Riesling) located in the Clare Valley. Isolates MA076 and MA002 did not form a clade with isolates MA089, MA101 and MA176 obtained from the same McLaren Vale vineyard (Fig 1A). The phylogenetic relationships between the McLaren Vale isolates MA089, SAPN01, MA101 and MA176, as well as the Clare Valley isolates C001, C002 and C003 suggested the presence of local recombination within specimens infecting the same vineyard. In agreement with previous population studies of *E*. *lata* [17–19], no clear grouping was observed based on either geographic location or host for the remaining isolates investigated (Fig 1A). These observations provide further evidence of gene flow between isolates infecting different hosts as previously reported by Travadon and Baumgartner [17].

Genetic reshuffling via sexual recombination and frequent gene flow between hosts has been suggested to prevent geographic and host differentiation in this species [17,18]. To estimate the degree of sexual recombination within the *E*. *lata* population, linkage disequilibrium (LD) was calculated for all biallelic SNPs present across the largest assembled chromosome (6.7 Mbp) of isolate TAS7 (Fig 1B). In recombining genomes, a decrease in linkage between two loci is expected as a function of the distance separating them. LD decay calculations using the squared correlation coefficient decreased to half of its maximum value between 100–150 bp (Fig 1B). Higher LD decays have been reported for well-studied sexually reproducing species such as *Neurospora crassa* (780 bp) [66]. Furthermore, LD decay distance as low as 110 bp has been observed in the obligately outcrossing mushroom *Schizophyllum commune* [67,68]. The short LD decay values observed for *E*. *lata* fall within the levels of sexually reproducing species and indicate that sexual reproduction and ascospore dispersal is the likely method of propagation for this population.

The lack of canonical mating-type regions has been reported for several members of the Xylariales [69] including *E*. *lata* strain UCREL1, where only a putative *MAT1-2-1* gene was annotated. Furthermore, this *MAT1-2-1* homolog was found to be unlinked to the orthologs of *sla2* and *apn2*, two genes that are normally linked to mating-type genes in ascomycetes species [70,71]. Examination of putative mating loci across the forty isolates in this study showed that all isolates contained a mating locus arrangement identical to UCREL1, with a putative *MAT1-2-1* gene unlinked to *sla2* and *apn2* (S3 Table). Mating type loci are known to be areas of recombination suppression in many fungal species [72]. LD was therefore investigated in all regions where a putative *MAT1-2-1* gene was found, as well as the locus of genes *sla2* and *apn2*. None of the regions showed signatures of high LD suggesting these regions do not determine the mating type of this species.

Inspection of the topology of the phylogenetic network showed the presence of a star-like tree characterized by long external branches (Fig 1A). Star-like phylogenies are consistent with a rapid expansion of a population following a bottleneck [73,74]. To further investigate the possibility of a recent bottleneck within the *E*. *lata* population, the minor allele frequency spectrum (MAF) (Fig 1C) and Tajima’s *D* statistic (Fig 1D) were estimated across this set of isolates. The MAF spectrum displayed a distribution towards rare alleles (Fig 1C), without a spectrum distortion that would be indicative of a very recent population bottleneck [75], while Tajima’s *D* statistic showed a tendency towards negative values (Fig 1D). Both measures are consistent with a rapid expansion of the Australian *E*. *lata* population. Establishment of *E*. *lata* in Australia has been previously proposed to have occurred through small founder population(s) [18]. The whole-genome population data is consistent with this theory, while further suggesting that the *E*. *lata* population is continuing to expand in Australia. It can be hypothesised that the recent significant growth experienced by the Australian wine industry has contributed to the rapid propagation of this species since its initial introduction into the country more than a century ago [1].

**Fig 2:**
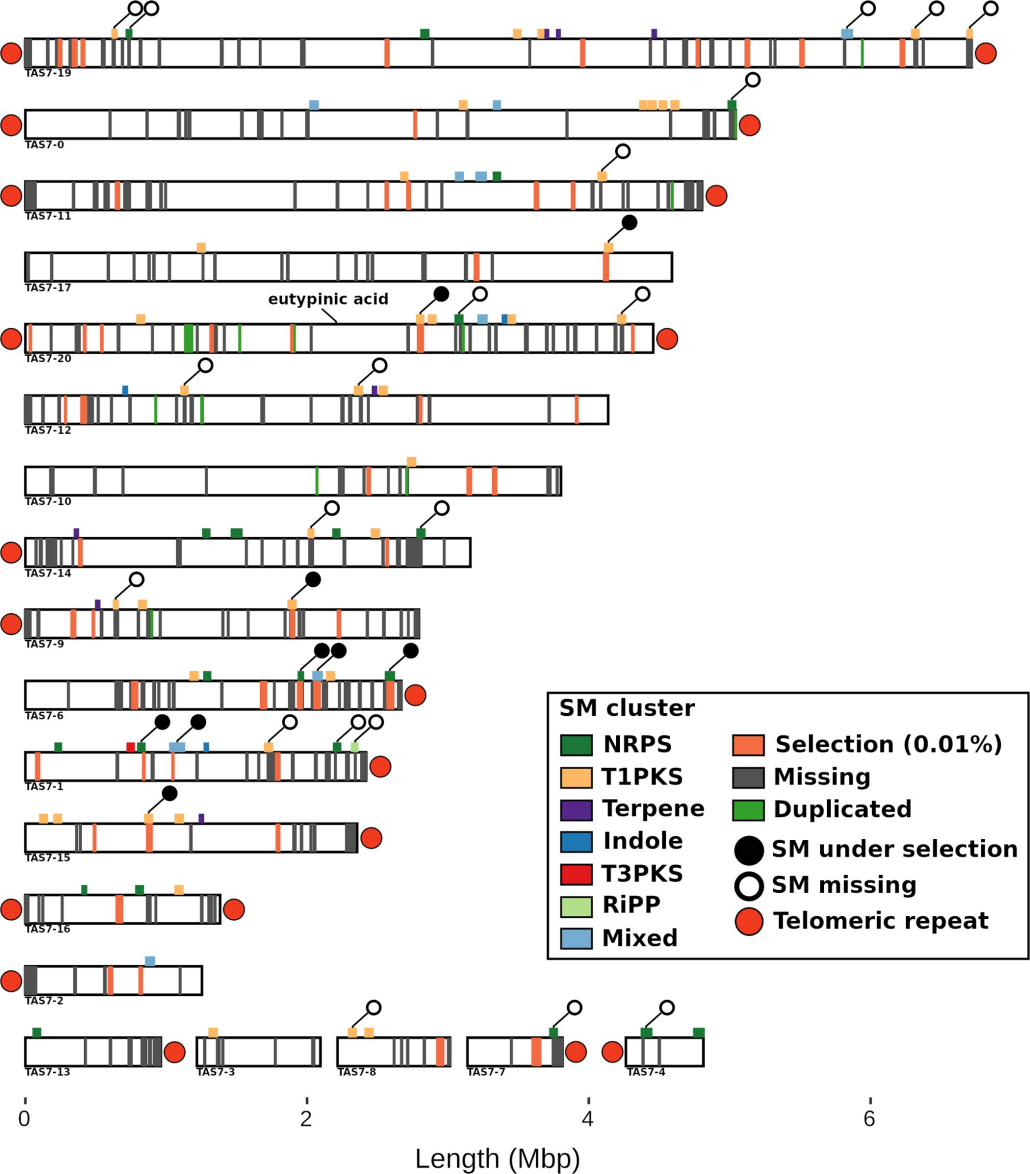
Karyotype representation of the genome assembly of *Eutypa lata* isolate TAS7. Predicted secondary metabolite (SM) clusters are shown above each contig with type of cluster indicated in the legend. Regions marked inside the contigs correspond to the following: (orange) top 0.01% *μ* scores representing signatures of selective sweep within the Australian population, (grey) missing genomic regions and (green) duplicated regions that overlap with CDSs present in at least one isolate. SM clusters under positive selection and missing in at least one sample are tagged with filled and empty circles, respectively. The genomic location of the previously reported eutypinic acid cluster [77] is marked.

The highly contiguous genome assemblies produced using long-read data allowed for the investigation of signatures of selective sweeps across the *E*. *lata* population. To avoid interferences of background selection and potential false positives arising from demographic history, a stringent percentile threshold of 99.99% was applied for the *μ* statistic (see Materials and Methods). After filtering, 53 genomic regions were identified as being under selection, which ranged from 3.6 to 40.7 kb in length and contained a total of 240 ORFs (Fig 2, S4 Table). The complement of ORFs located within these selective sweep regions were enriched (p-value: 7.74e-10) for genes predicted to comprise members of several secondary metabolite clusters (S4 Table, Fig 2). The role of secondary metabolites in the lifecycle of *E*. *lata* is poorly understood, however these clusters likely have diverse roles including pathogenicity, the inhibition of competing microorganisms, host adaptation and dealing with environmental stressors, as previously reported in other fungal species [76].

Acetylenic phenols and chromene secondary metabolites have been proposed as the main phytotoxins produced by *E*. *lata* [9], however the specific biosynthetic clusters that are involved in the production of these compounds have not been experimentally confirmed. Recently, a putative cluster responsible for the synthesis of compounds, such as eutypine, that contain a 1,3-enyne moiety was identified and confirmed in an *Aspergillus* sp. [77]. The genome of *E*. *lata* strain UCREL1 contains a similar putative gene cluster, which has been previously suggested as being responsible for the production of eutypine [77]. It was possible to identify all nine genes that belong to this cluster in each of the forty isolates sequenced in this study (Fig 2). However, this cluster was not located within any region proposed to have undergone a selective sweep (Fig 2), indicating that the eutypinic cluster was not under recent positive selection.

Regions affected by selective sweeps also encoded proteins playing a role in transmembrane transport (Zn/Fe and amino acid permeases, drug and metal resistance and sugar and vitamin MFS transporters), degradation of host cell wall (CAZymes GH28, GH55, GH93, AA3 and AA7), protein degradation (MEROPS S08A, S10, S12 and M20D) and regulatory pathways, amongst others (S4 Table). The broad range of gene functions suggests that the *E*. *lata* population is adapting to both abiotic factors and potentially host genotypes. By using previously published meta-transcriptomic data [62] and a multi-species reference genome (see Materials and Methods) including the assembly of isolate TAS7, 166 of these genes were shown to be expressed in-planta (TPM > 20) (S4 Table), suggesting they play a role during the symptomatic growth of this species in *V*. *vinifera*. Comparative analysis with established *E*. *lata* populations from other continental regions such as Europe and California will help to elucidate if there is divergence in selection due to environmental factors and diversity of hosts.

Comparison of the high-quality genome assemblies revealed significant intraspecific genomic plasticity. Synteny analyses showed the presence of extensive intra- and inter-chromosomal rearrangements, including large translocations, duplications and deletions of gene encoding regions (S1 Fig).

The genome architecture of fungal plant pathogens is known to be highly diverse [78] with intraspecific differences in genome size and architecture previously reported for several filamentous Ascomycetes [79,80]. Genomic loci that are rich in DNA repeats and/or transposable elements have been shown to frequently coincide with these breakpoints in genomic synteny [80–83], and are thought to evolve at faster rates, as well as harbour virulence-related genes [78].

To investigate the involvement of repetitive elements in the observed structural rearrangements between isolates of *E*. *lata*, the location of repetitive loci was assessed relative to breakpoints in synteny between the four isolates. Inspection of individual breakpoints in synteny between isolates 511-17/MA101 (S1A Fig) and TAS7/MA101 (S1B Fig) showed that 19 of 32 (511–17) and 27 of 41 (TAS7) (S1A Fig), as well as 21 of 27 (TAS7) and 18 of 25 (MA101) (S1B Fig) synteny breakpoints, respectively, were associated with repeat regions and LTR retrotransposons, suggesting that these elements are also providing the means for alterations in genome architecture in this species.

While most of the genetic diversity of *E*. *lata* is thought to arise from sexual recombination, these structural rearrangements may play a crucial role in the diverse pathogenicity reported [6] through gain and loss of virulence related genes [80]. Chromosomal rearrangements have also been linked with suppressed recombination [84], however, the short LD-decay values estimated in this population (Fig 1B) suggest these rearrangements are not largely affecting recombination.

To investigate how these genomic architectural differences impact the gene content of this population, the extent of the core and pan-genome was estimated using the predicted ORFs of all forty isolates. Pan-genome analysis indicated that 85% of predicted ORFs in TAS7 are shared across the population, with the *E*. *lata* core (ORFs present in all isolates) genome comprising 12533 ORFs. The soft-core genome (ORFs present in 95% of isolates), which allows for the presence of potentially missing or fragmented genes, was composed of 13184 ORFs (S2 Fig). From the pan-genome, the shell (3 to 37 isolates) and cloud (≤ 2 isolates) genomes were composed of 4178 and 2331 ORFs, respectively (S2 Fig).

Genes within the shell and cloud represent a flexible genome that may reflect the individual lifestyle and adaptation of these isolates and captures sequences affected by structural variations. Enrichment analysis of Pfam domains associated with these isolate-specific genes showed significantly enriched domains in both the shell and cloud genomes that were associated with secondary metabolism, including polyketide synthases and cytochrome P450s (S5 Table), suggesting variability in the secondary metabolite production potential between these isolates.

While the abundance of specific classes of predicted secondary metabolite clusters across the four long-read assemblies were similar (S6 Table), reference-based mapping (against TAS7) of 39 isolates showed that only 62 out of the 82 predicted clusters observed in TAS7 were shared across these forty isolates (Fig 2).

Previous phenotypic screens of *E*. *lata* have demonstrated significant variability in the production of key metabolites between isolates [8,85,86], as well as significant variation in levels of pathogenicity [6,87–89]. When combined with the results of the pan-genome and reference-based analyses, it is evident that the metabolite production potential within *E*. *lata* is highly variable and could explain the broad differences in pathogenicity reported in several studies [6,87–89]. Future screens using untargeted metabolomics and the development of novel metabolomic methods [90] will be needed to confirm the diversity of metabolites produced, and how these compounds correlate with differences in pathogenicity and lifestyle of this species.

Variations in mycelium growth has been previously reported for 25 of the *E*. *lata* isolates included in this study [6]. Mycelial growth and colonization within the woody tissues of *V*. *vinifera* is considered a determinant of disease severity, with important implications for disease management, often requiring removal of tissue to avoid complete colonization and death of the infected grapevine. To investigate potential associations between genomic elements and the reported phenotypes, a k-mer-based GWAS methodology was applied [59]. Short-read sequences were used to assemble a k-mer database that was subsequently correlated against previously published phenotypic data concerning mycelial recovery of the sequenced isolates of *E*. *lata* within canes of *V*. *vinifera* [6]. The k-mer based methodology allowed for reference-free association of a broad range of genetic variants, including structural variations, that are not usually observed with conventional SNP-based GWAS methodologies.

**Fig 3:**
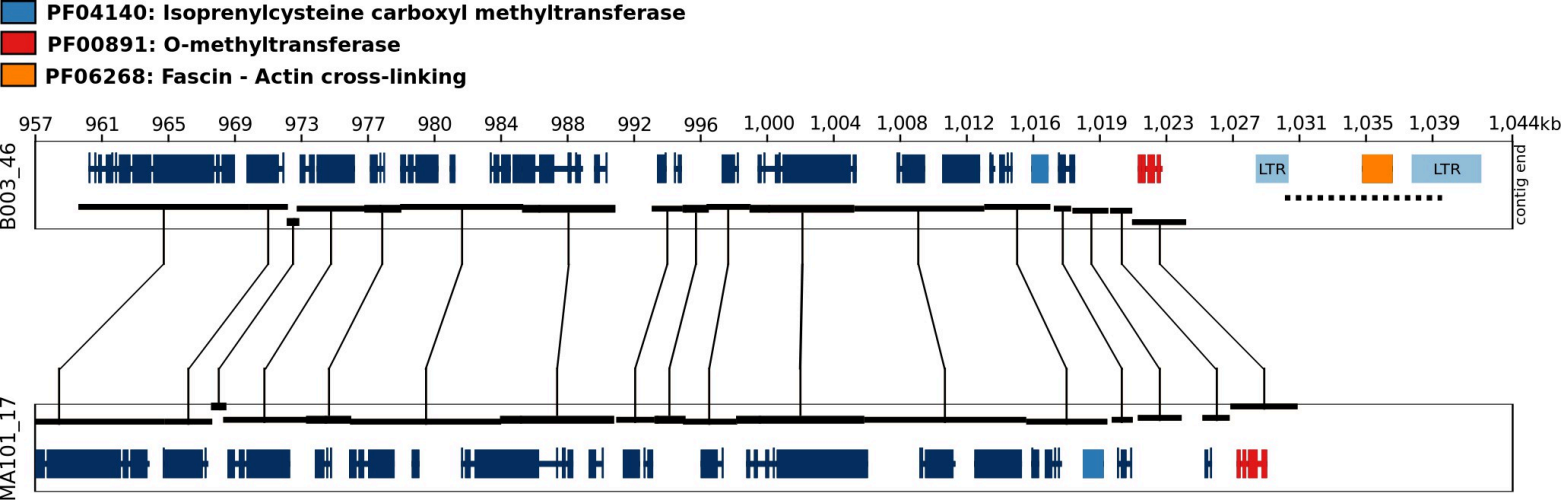
Genomic location of the 20 most significant k-mers associated with previously published phenotype data of 25 *Eutypa lata* isolates [6]. Synteny blocks between isolates B003 (k-mer containing isolate) and MA101 (absence of k-mers) are represented with black linked boxes. The mapping genomic location of long-reads containing the 20 most significant k-mers (p-value 6.65e-7) in the genome of isolate B003 is denoted with a dotted line. Pfam domains for neighbouring genes are indicated in the legend. The presence/absence of these kmers and the phenotype data are available in S7 Table.

Genomic locations that could be potentially linked to differences in mycelial recovery were defined by selecting the 20 most significantly associated k-mers, followed by mapping the k-mer associated long-reads back to the genome assembly of the isolate (Fig 3, S7 Table). Inspection of k-mer associated mapping locations showed that all reads were localised to a single genomic region that was predicted to encode a protein with an actin cross-linking domain (Fig 3), suggesting a possible role in actin-crosslinking and hyphal growth. In isolate B003, this gene is flanked by predicted LTR elements (Fig 3). Inspection of this region in isolate MA101 (non-k-mer containing isolate, S4 Table) showed a complete absence of the region, including the predicted ORF and flanking LTR elements (Fig 3). Due to the presence of repeat elements and the observed relationship between these elements and genomic synteny breakpoints (S1 Fig), it is likely that this region has been subjected to LTR-induced structural rearrangement in several of the isolates investigated. These preliminary results will need to be confirmed using a larger phenotype sample size as commonly employed in standard GWAS analyses. Genetic transformation of *E*. *lata* using previously reported protoplast-based methods [91] will also be required to confirm the functionality of this genomic locus.

## 4. Conclusions

In this study we performed genome sequencing and comparative analyses of forty *E*. *lata* isolates sourced from diverse grape-growing regions across Australia, representing the first whole-genome population study of this important agricultural pathogen. The genetic diversity of this population showed a high degree of gene-flow and sexual recombination between isolates sourced from different hosts and geographic locations as well as signs of recent demographic expansion. Inspection of signatures of selective sweeps, repeat-mediated chromosomal rearrangements and pan-genomic elements revealed a highly dynamic secondary metabolite production potential that could have important implications for the pathogenicity and lifestyle of this species. K-mer based GWAS analysis identified a locus associated with mycelia recovery in canes of *V*. *vinifera* that will require further investigations. This study also provides a publicly available dataset of sequencing reads and genome assemblies including four isolates assembled using long-read technology. These genomic resources will be of high importance for future investigations aiming to understand the physiology, pathogenesis, and global genetic diversity of *E*. *lata*.

## Supporting information

S1 Fig Synteny between the genome assemblies of four *Eutypa lata* isolates represented as circos plots. Synteny blocks are coloured based on the reference sequence and query assembly is represented in grey. Query contigs have been ordered based on synteny length to reference. Contig IDs are labelled A) Genome synteny between *E*. *lata* isolate MA101 and isolate 511–17 and B) *E*. *lata* isolate MA101 and isolate TAS7. Repeat elements neighbouring (< 2 kb) synteny breakpoints are shown, excluding repeats located in contig ends. C) Genome synteny between isolates TAS7, B003, MA101 and 511–17.
(TIF)

S2 Fig Gene-based pan-genome analysis of forty *Eutypa lata* isolates. (TIFF)

S1 Table Isolation details of the 40 *Eutypa lata* isolates included in this study. (XLSX)

S2 Table Genome assembly and annotation statistics for the 40 *Eutypa lata* isolates included in this study. (XLSX)

S3 Table Locus of putative *MAT1-2-1*, *sla2* and *apn2* genes in the 40 isolates investigated. (XLSX)

S4 Table Functional annotation and expression of genes located within regions under selective sweep. (XLSX)

S5 Table Enriched Pfam domains in shell and cloud genomes. (XLSX)

S6 Table Predicted secondary metabolite gene clusters in the long-read assembly of four *Eutypa lata* isolates. (XLSX)

S7 Table Presence/absence of the top significant k-mer and mycelium recovery phenotype for 25 *Eutypa lata* isolates. (XLSX)

S1 Data Annotation files for 39 *Eutypa lata* isolates in GFF3 format. (ZIP)

S2 Data Numerical data underlying graphs presented in Fig 1. (XLSX)
