# Supplementary figures and images for: Population genomics of the grapevine pathogen Eutypa lata reveals evidence for population expansion and intraspecific differences in secondary metabolite gene clusters
Source: PLoS Genet. 2022 Apr 1;18(4):e1010153. doi: 10.1371/journal.pgen.1010153 (PMC9007359; doi:10.1371/journal.pgen.1010153)

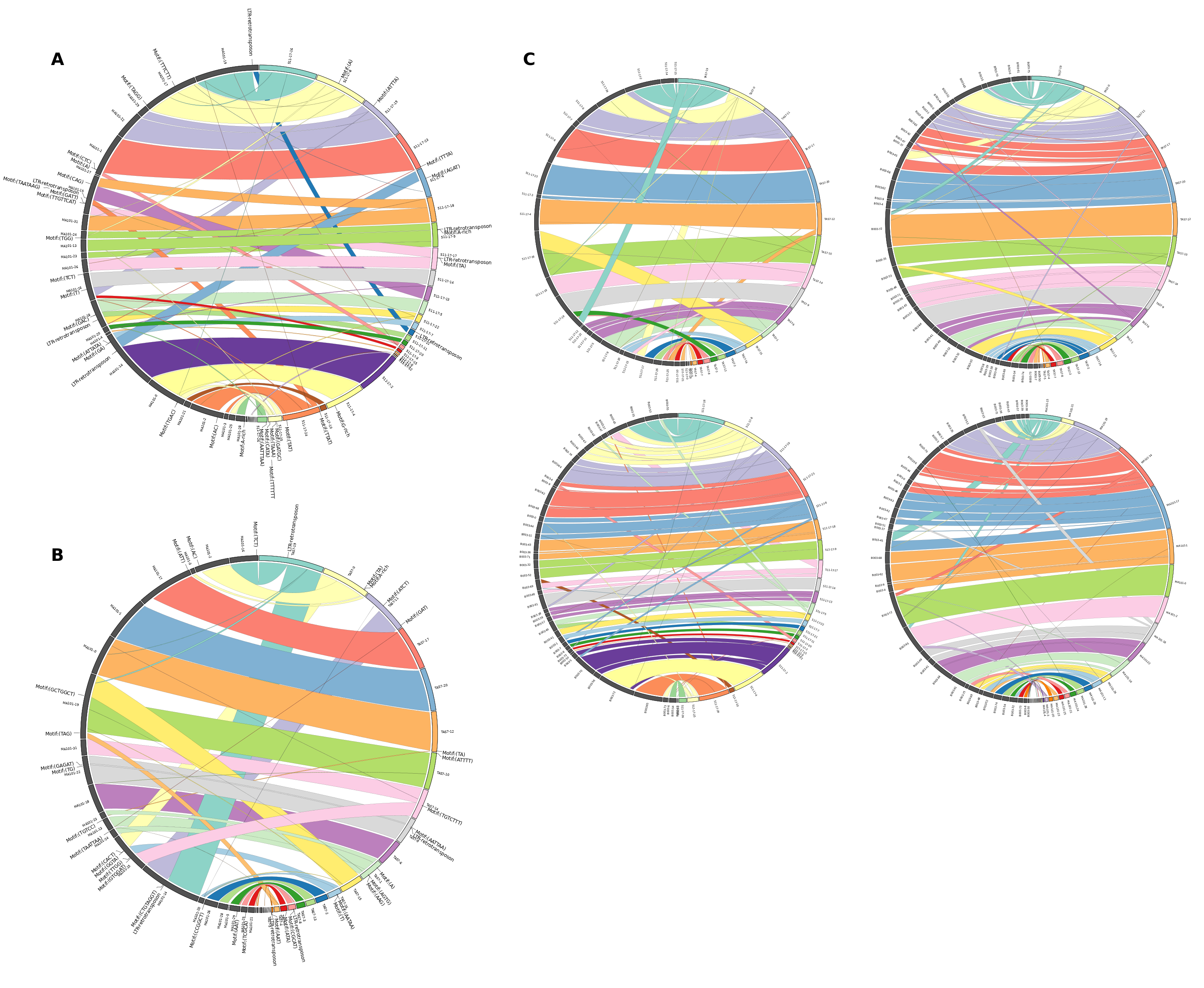

Supplement: S1 Fig — Synteny blocks are coloured based on the reference sequence and query assembly is represented in grey. Query contigs have been ordered based on synteny length to reference. Contig IDs are labelled A) Genome synteny between E. lata isolate MA101 and isolate 511–17 and B) E. lata isolate MA101 and isolate TAS7. Repeat elements neighbouring (< 2 kb) synteny breakpoints are shown, excluding repeats located in contig ends. C) Genome synteny between isolates TAS7, B003, MA101 and 511–17. (TIF) [file pgen.1010153.s001.tif]

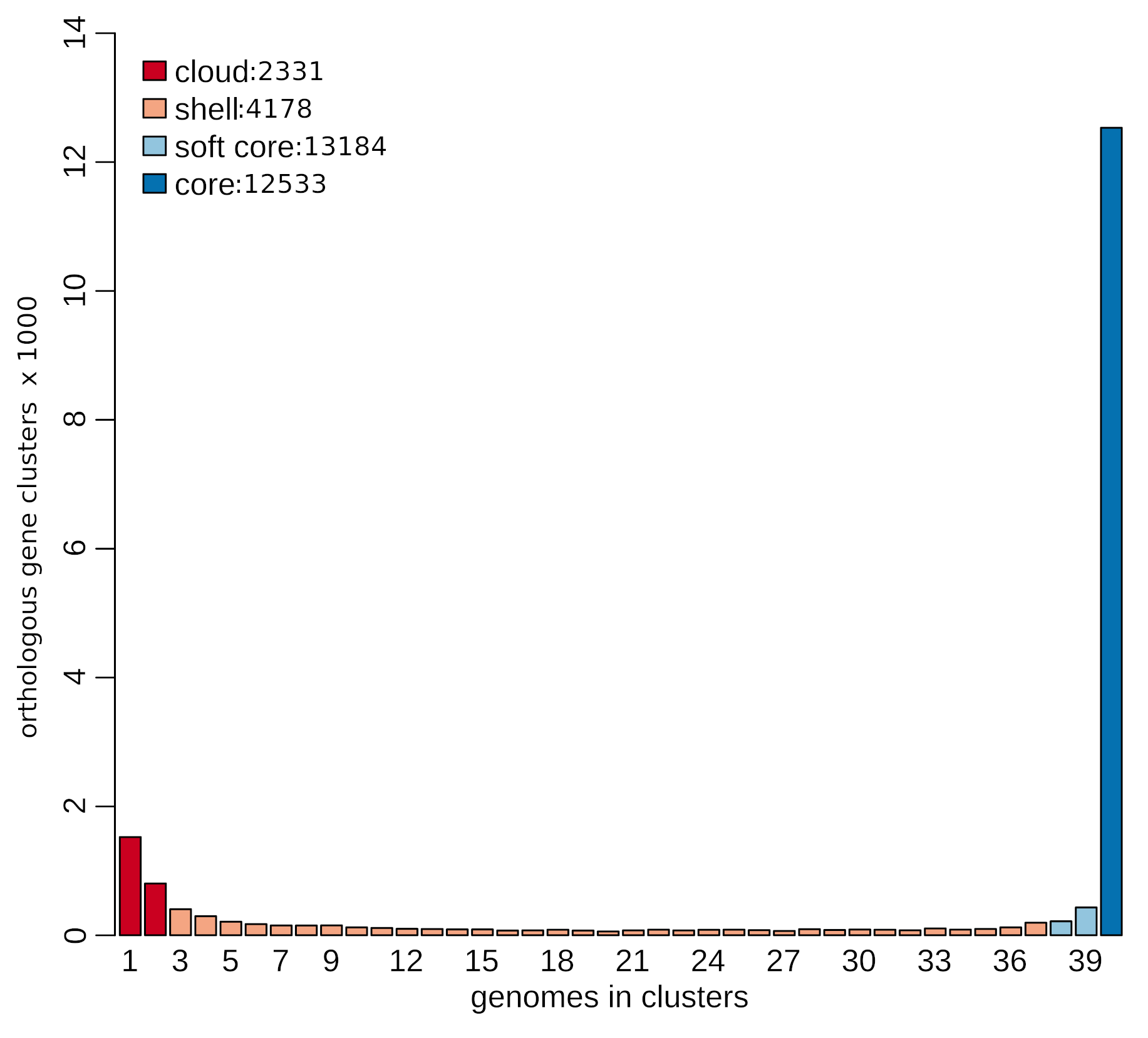

Supplement: S2 Fig — (TIFF) [file pgen.1010153.s002.tiff]
